# A Pressing Emergency in Oncology: A Case Series of Patients With Posterior Reversible Encephalopathy Syndrome

**DOI:** 10.7759/cureus.75028

**Published:** 2024-12-03

**Authors:** Vikram L Narasimhan, P Pavan Kumar

**Affiliations:** 1 Department of Critical Care Medicine, Vydehi Institute of Medical Sciences and Research Centre, Bangalore, IND; 2 Department of General Medicine, Vydehi Institute of Medical Sciences and Research Centre, Bangalore, IND

**Keywords:** brain capillary leak syndrome, chemotherapy-related toxicity, hyper-perfusion encephalopathy, posterior reversible encephalopathy syndrome in non hypertensives, posterior reversible encephalopathy syndrome in oncology, reversible posterior leukoencephalopathy syndrome (rpls)

## Abstract

Background: The defining characteristic of posterior reversible encephalopathy syndrome (PRES) is a reversible, predominantly vasogenic edema of the white matter, particularly affecting the parenchyma supplied by the posterior circulation. PRES is most commonly associated with hypertension. We present a case series of seven normotensive patients diagnosed with cancer who had posterior reversible encephalopathy syndrome.

Materials and methods: This series of retrospective cases includes seven patients hospitalized between August 2022 and October 2024, all with a primary oncological condition and diagnosed with PRES either at admission or during their hospital stay. PRES was established according to the diagnostic criteria outlined in the 2012 Berlin Study. Our study concentrated on clinical characteristics, including underlying disease, triggering clinical events, chemotherapy agents, and outcome measures such as reversibility, functional status, and mortality.

Results: The median age of patients in this study was 48 years. No patient exhibited significantly elevated blood pressure during their inpatient stay. Altered consciousness with seizures was the primary initial manifestation in most patients, followed by headache. The predominant observation on the MRI was T2 flair hyperintensity in the posterior circulation. All subjects attained nearly full neurological recovery by 28 days, regardless of steroid therapy. The 90-day all-cause mortality rate was 14% (one out of seven patients). There were no fatalities attributable to PRES.

Conclusion: Posterior reversible encephalopathy syndrome is a neurological emergency that may evade the discerning attention of the attending physician. Owing to the extensive range of clinical features and MRI findings, the list of differential diagnoses is substantial. The reversibility of symptoms, while not always applicable, is predominantly certain, as demonstrated in our case series. We must extend our focus beyond conventional risk variables such as hypertension to consider additional clinical insults. Delayed diagnosis may lead to worse neurological outcomes.

## Introduction

Hinchey et al. (1996) described posterior reversible encephalopathy syndrome (PRES) as a clinico-radiological syndrome characterized by a reversible, predominantly vasogenic edema of the white matter with a predilection for parenchyma supplied by the posterior circulation [1]. Pathogenesis revolves around multiple postulated theories, including vasogenic theory, cytotoxic theory, immunogenic theory, and neuropeptide theories, that finally culminate into endothelial dysfunction at the blood-brain barrier, leading to cerebral edema [2]. Better sympathetic innervation of the anterior circulation protects the brain from sudden changes in cerebral blood flow and failed autoregulatory mechanisms. Hence, posterior circulation is more prone to PRES [3]. 

Comorbid diseases such as hypertension, renal failure (acute or chronic), organ transplantation, cancer, infection, autoimmunity, eclampsia, and immunosuppression or chemotherapy are all correlated with PRES in the same order of prevalence. PRES can manifest with a diverse array of clinical features, including seizures, headaches, vomiting, visual disturbances, decreased levels of consciousness, and motor deficits [4]. The differential diagnosis of PRES encompasses hypertensive encephalopathy, eclampsia, reversible cerebral vasoconstriction syndrome, encephalitis, and cerebral venous sinus thrombosis. The management of PRES involves controlling blood pressure in hypertensive patients, managing seizures, discontinuing offending medications, and correcting dyselectrolytemia. The role of steroids remains a subject of controversy.

This study seeks to elucidate the clinical characteristics, encompassing magnetic resonance imaging, cerebrospinal fluid analyses, and electroencephalogram data, in patients with PRES, with an emphasis on reversibility.

## Materials and methods

In this series of retrospective cases, we identified seven patients having a prior diagnosis of cancer who were admitted to wards or intensive care units of Vydehi Institute of Medical Sciences and Research Centre, a tertiary care facility located in Bangalore, Karnataka, India, from August 2022 to October 2024. This research was approved by the Vydehi Institutional Ethics Committee (VIEC/2024/APP/83, dated 20th November 2024). These patients were diagnosed with PRES either at presentation or during their hospital stay, according to the clinico-radiological criteria established in the Berlin PRES study of 2012, as detailed below [4].

1) Early onset of PRES-specific clinical symptoms, such as headache, nausea or vomiting, visual abnormalities, epileptic seizures, and other focused impairments.

2) Vasogenic edema foci, which can vary in distribution and intensity, together with or without foci of diffusion restriction, hemorrhagic symptoms, or any other imaging features suggestive of PRES.

3) The existence of certain toxic correlations that could cause mild to severe arterial hypertension and/or endotheliotoxic consequences.

4) Other potential causes of vasogenic edema, such as meningoencephalitis, space-occupying lesions, cerebral venous sinus thrombosis, and hypoxic-ischemic encephalopathy, were excluded through clinical history, signs, cerebrospinal fluid analysis, MR venography, and MRI with contrast when deemed necessary.

Data regarding patients' demographic characteristics, clinical manifestations, co-morbidities, histopathological cancer diagnoses, staging at presentation, and chemotherapy details, were extracted from electronic medical records. Data from brain MRI and CT scans, cerebrospinal fluid analyses, electroencephalogram results, antiepileptic treatment, and intensive care management were collected. Blood pressure measurements at the time of admission and during the hospital stay were extracted from the medical records. In an acute setting, blood pressure was defined by AHA guidelines, which categorized markedly increased inpatient blood pressure or accelerated hypertension as a measurement exceeding 180/110 mmHg [5]. All patients underwent MRI scans utilizing a 16-channel system, 1.5 TESLA SIEMENS MAGNETOM SEMPRA SCANNER. An experienced neurologist and a radiologist, both blinded to each other's assessments, independently confirmed the diagnosis of PRES on MRI, supplementing the earlier diagnosis. Patients included in the case series were monitored during subsequent outpatient department visits or through telephonic follow-ups to assess neurological status and mortality. Neurological outcomes were evaluated using the Modified Rankin Scale (mRS) (Table 1), ranging from zero to six at both diagnosis and 28 days post-admission [6]. All-cause mortality was assessed 90 days post-admission.

**Table 1:** The Modified Rankin Scale

Score	Description
0	No symptoms at all
1	No significant disability despite symptoms; able to carry out all usual duties and activities
2	Slight disability; unable to carry out all previous activities but able to look after own affairs without assistance
3	Moderate disability; requiring some help but able to walk without assistance
4	Moderately severe disability; unable to walk without assistance and unable to attend to own bodily needs without assistance
5	Severe disability; bedridden, incontinent and requiring constant nursing care and attention
6	Dead

## Results

This retrospective case series included seven patients, comprising four females (57%) and three males (43%). The median age of this cohort was 48 years, with a range of 24 to 66 years. No patient exhibited accelerated hypertension.

Two patients presented with hematological malignancies, whereas five patients exhibited solid organ malignancies.

Seizure (86%) with altered consciousness (86%) was the primary initial manifestation in most patients, followed by headache (43%). One patient (case 1) had a neurological deficit characterized by left hemiparesis. No patient experienced visual disturbances. A prevalent precipitating factor for PRES identified in all patients was the presence of malignancy itself. Five patients were undergoing active chemotherapy, while one was receiving immunotherapy with Nivolumab. Additionally, renal dysfunction was noted as another precipitating factor in one patient.

Table 2 provides a detailed description of the aforementioned clinical characteristics.

**Table 2:** Baseline Clinical Characteristics Upon Presentation With Posterior Reversible Encephalopathy Syndrome F: Female, M: Male

Demographics	Case 1: 53/M	Case: 2 66/F	Case 3: 24/M	Case 4: 35/M	Case 5:34/F	Case 6: 55/F	Case 7: 48/F
Premorbid diagnosis	Retroperitoneal spindle cell carcinoma (Stage 1b)	Serous papillary endometrial carcinoma (Stage 3)	Chronic myeloid leukemia	Mucinous carcinoma, rectum (Stage 2)	T-cell acute lymphoblastic leukemia	Invasive ductal carcinoma breast (Stage 4)	Squamous cell carcinoma cervix (Stage 4)
Hypertension at presentation	No	No	No	No	No	No	No
Other comorbidities	No	Hypothyroidism	No	No	No	No	No
Seizures	Yes	Yes	Yes	No	Yes	Yes	Yes
Headache	No	No	Yes	Yes	Yes	No	No
Altered mentation	Yes	Yes	Yes	No	Yes	Yes	Yes
Visual disturbances	No	No	No	No	No	No	No
Other neuro deficits	Left hemiparesis	No	No	No	No	No	No
Chemotherapy at presentation	No active chemotherapy	Nivolumab	Imatinib	6th cycle: capecitabine, oxaliplatin	Maintenance phase: 6-mercaptopurine, methotrexate	4^th^ cycle: Paclitaxel-Carboplatin	2nd cycle: Paclitaxel Carboplatin
Precipitating factors	Malignancy	Malignancy, Immunotherapy	Malignancy, Chemotherapy	Malignancy, Chemotherapy	Malignancy Chemotherapy	Malignancy, Chemotherapy	Malignancy, Chemotherapy, Renal dysfunction

All seven patients underwent an MRI of the brain, with or without the use of contrast agents. Patchy T2-weighted hyperintensities in the parieto-occipital regions were observed in 86% of patients, characterized by the absence of diffusion restriction, blooming on a gradient, and contrast enhancement, representing the most prevalent finding of vasogenic edema. Other less involved lobes included the frontal (57%), cerebellar (28%), and temporal (14%) regions. The sole patient in the series (Case 1) exhibiting left hemiparesis demonstrated diffusion restriction in the thalamus on MRI.

**Figure 1:**
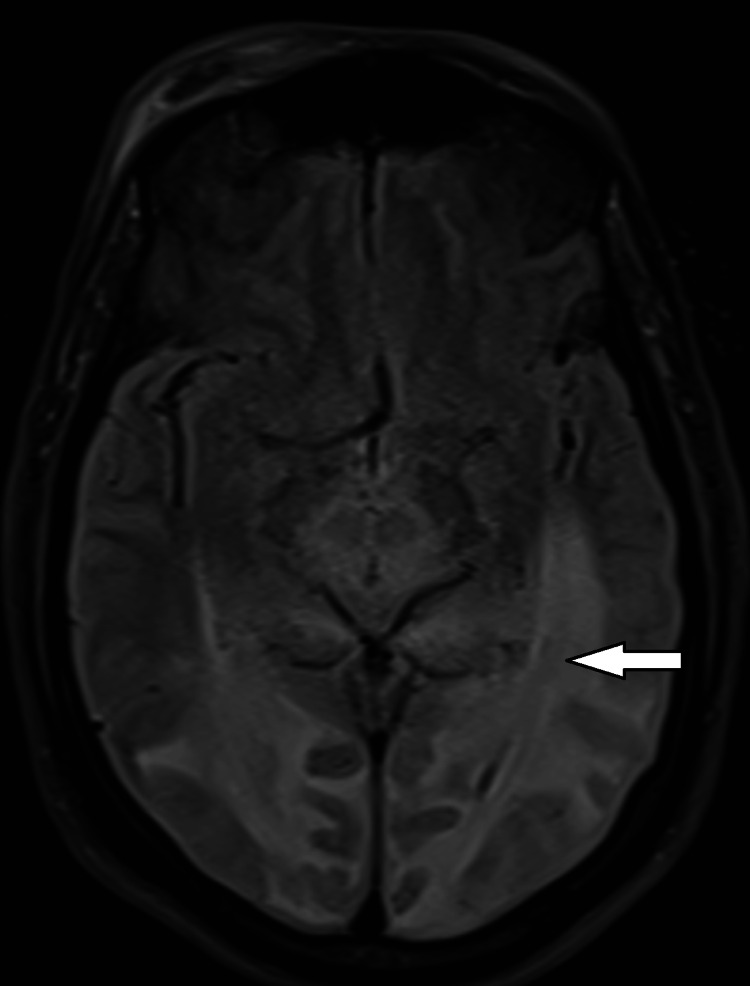
T2 FLAIR Sequence on Magnetic Resonance Imaging in Case 1 The white arrow indicates hyperintensities, representing vasogenic edema. FLAIR: Fluid-attenuated inversion recovery

**Figure 2:**
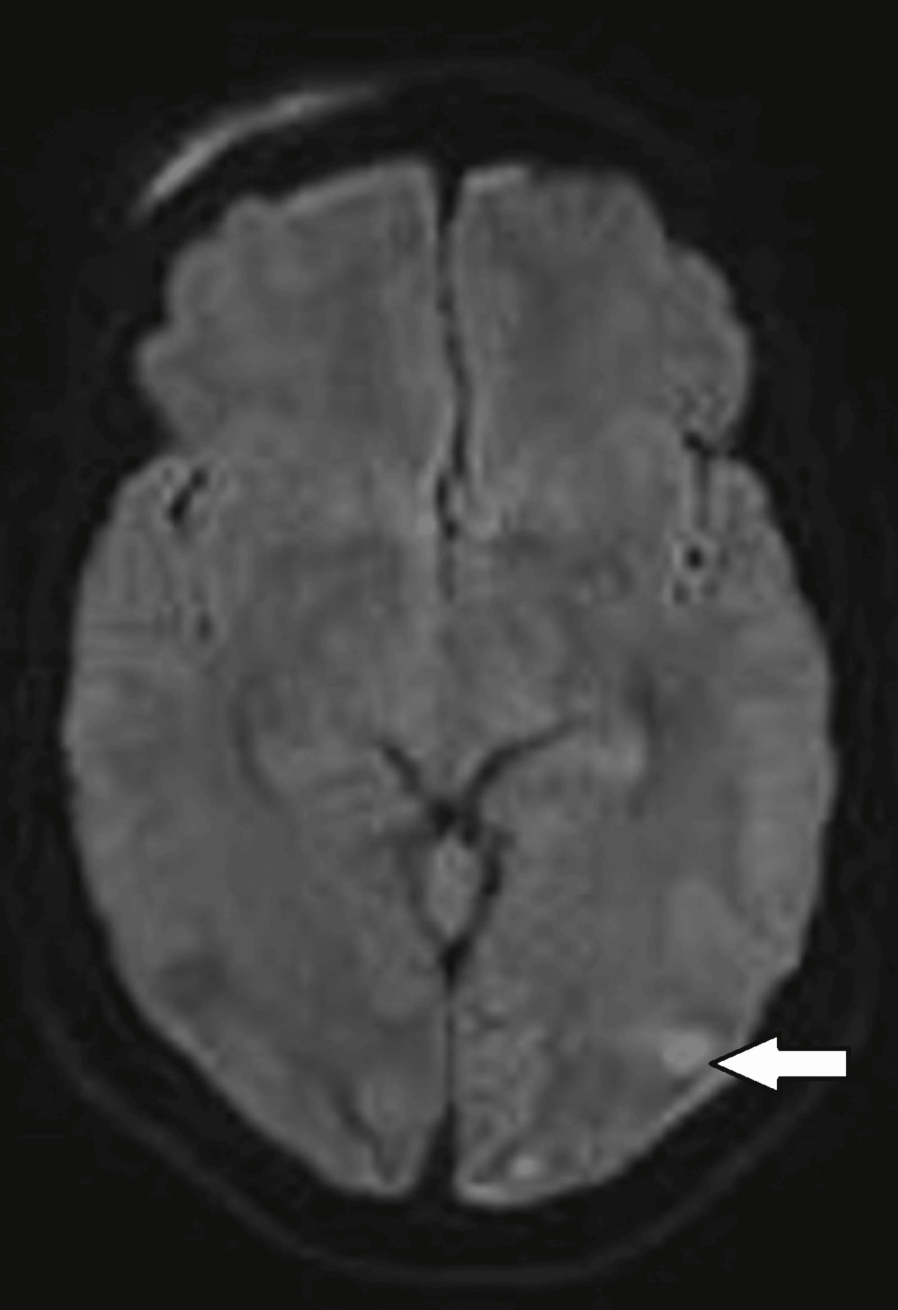
DWI Sequence on Magnetic Resonance Imaging in Case 1 The white arrow represents a diffusion restriction suggesting ischemia DWI: Diffusion weighted imaging

**Figure 3:**
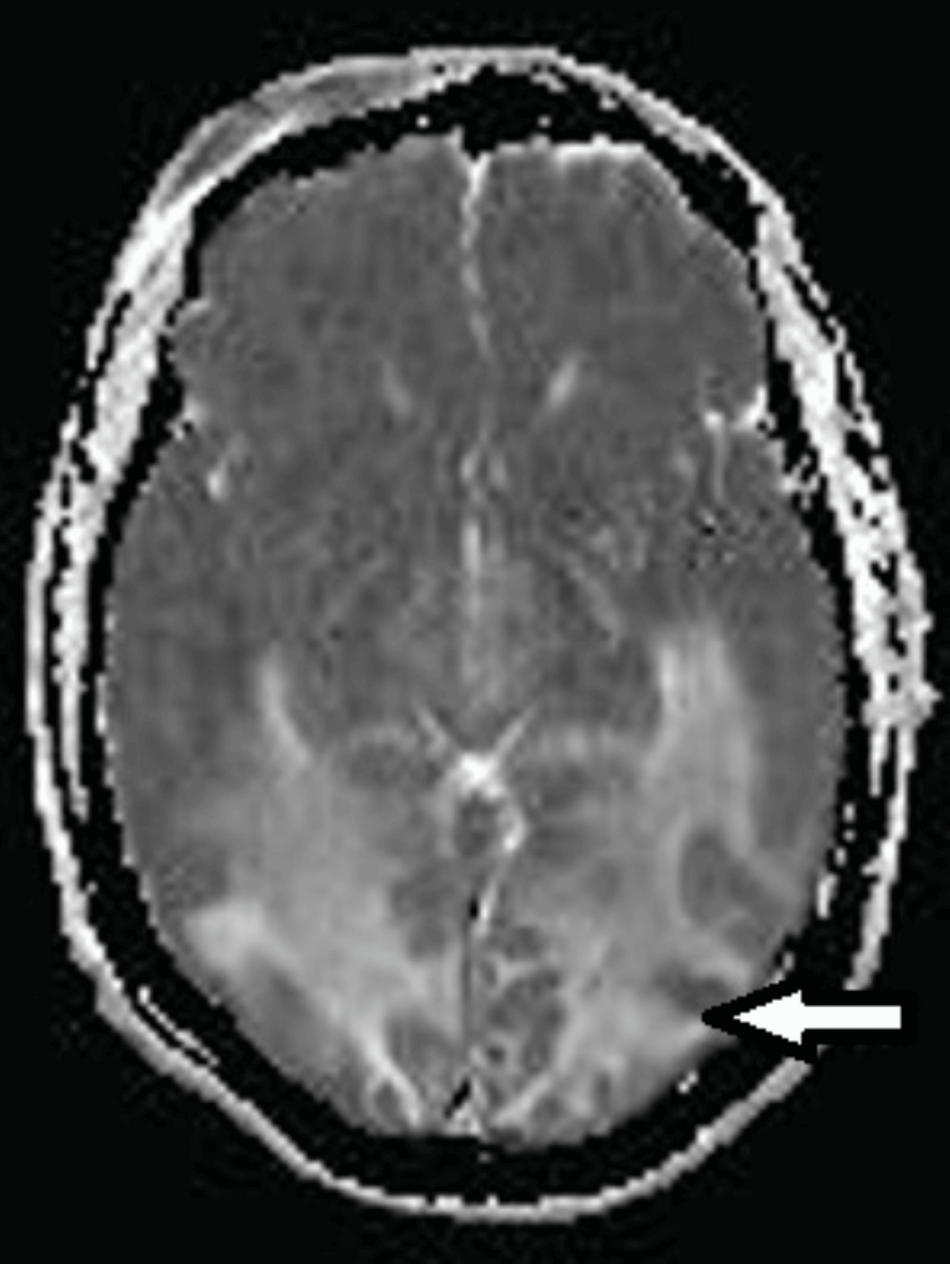
ADC Sequence on Magnetic Resonance Imaging in Case 1 The white arrow represents hypointensity, corresponding to the diffussion restriction on diffussion-weighted imaging. ADC: Apparent Diffussion Coefficient

MRI images in case 1 are shown in Figures 1-3.

The predominant EEG finding was diffuse slowing of background frequencies, indicative of encephalopathy.

Analysis of cerebrospinal fluid was conducted in only two patients. Case 2 exhibited a normal cell count (1 lymphocyte) and glucose level (55 mg/dL), alongside elevated protein levels (116 mg/dL). Case 3 with chronic myeloid leukemia exhibited elevated protein levels of 100 mg/dL and presented with eight atypical cells. Infectious pathogens were ruled out.

Patients exhibiting seizures were administered antiepileptic medications, first with levetiracetam as the primary therapy, followed by the addition of sodium valproate and carbamazepine in cases of status epilepticus. Three patients received a high dose of 1 gm methylprednisolone for a duration of five days at the discretion of the treating physician.

Table 3 presents a comprehensive evaluation of the patient, including treatment and outcomes.

**Table 3:** Investigations, Treatment and Outcomes F: Female, M: Male, MP: Methyl Prednisolone, MRI: Magnetic Resonance Imaging, CSF: Cerebrospinal Fluid, mRS: Modified Rankin Scale, * 28 days post-admission, NA: Not applicable

Parameters	Case 1: 53/M	Case 2: 66/F	Case 3: 24/M	Case 4: 35/M	Case 5: 34/F		Case 6: 55/F	Case 7: 48/F
Dyselectrolytemia	No	No	No	No	No	No		No
Renal dysfunction	No	No	No	No	No	No		Yes
CSF Analysis	NA	Elevated proteins	Pleocytosis, elevated proteins.	NA	NA	NA		NA
MRI lobes involved	Frontal Occipital Parietal	Occipital Parietal Cerebellar	Frontal Parietal Occipital	Occipital Parietal	Occipital Parietal	Cerebellar Temporal Frontal		Parietal Occipital Frontal
High-dose MP	Yes	Yes	Yes	No	No	No		No
mRS at admission	5	4	3	1	1	2		1
mRS at 28 days*	2	0	0	0	0	0		0
90-day mortality	Survived	Survived	Expired	Survived	Survived	Survived		Survived

Two patients (cases 1 and 2) required intubation due to significant neurological impairment with Modified Rankin Scale (mRS) scores of five and four, respectively. At 28 days, 86% of participants achieved complete neurological recovery, as indicated by a Modified Rankin Scale (mRS) score of zero.

At 90 days, 86% of the patients (n = six) were reported to have survived. Case 3 with chronic myeloid leukemia (CML) succumbed during a subsequent admission due to sepsis and the progression of the primary malignancy.

## Discussion

Neurological manifestations resulting from acute increases in blood pressure and subsequent loss of cerebral autoregulation in hypertensive individuals have been documented in the literature since the 1980s [3,7]. However, the notion that PRES occurs solely in hypertensive patients was first challenged by Hinchey and colleagues [1] and later supported by case series involving exclusively non-hypertensive individuals [8]. The findings of our study were consistent, showing that all participants exhibited normal blood pressure, in contrast to several other studies where the majority of the population displayed accelerated hypertension [4,9]. The median age of this cohort of patients was 48 years, indicating that PRES can manifest across various age groups. Besides hypertension, renal dysfunction was identified as the second most common trigger for PRES in the study by Liman et al. [4]. Our study involved one patient in whom renal dysfunction was identified as a potential trigger. Malignancy has been recognized as a significant comorbidity linked to PRES in non-hypertensive patients, in addition to traditional risk factors, as demonstrated by various studies [1,4,8]. The Berlin PRES study reported malignancy prevalence rates of up to 32% in patients with PRES [4]. Chemotherapeutic agents such as taxanes, platinum derivatives, vinca alkaloids, antimetabolites, anthracyclines, angiogenic inhibitors, folate antagonists, and immunosuppressants have been linked to PRES [10-12]. Limited case reports indicate an association between checkpoint inhibitors, such as nivolumab, used in endometrial and esophageal carcinoma, and PRES [13,14]. These findings align with our study as well. The occurrence of clinical features was consistent with findings from previous studies [9]; however, a few atypicalities were noted, including the absence of visual disturbances in our cohort.

Numerous studies have reported extensive parenchymal involvement, even in areas not supplied by the posterior circulation [4,8]. Our study also included 57% of patients who were involved in bilateral frontal lobes. This demonstrates that PRES can influence the anterior circulation as well. Elevated cerebrospinal fluid protein is frequently observed in patients with posterior reversible encephalopathy syndrome, as seen in our patient, and may indicate disruption of the blood-brain barrier. CSF pleocytosis, although infrequent, may occur, and its presence should not rule out the diagnosis of PRES [15].

Ismal FS et al. demonstrated in their study that clinical and MRI reversibility was observed in only 58% of patients, while mortality attributed to PRES was noted in 13% of patients [9]. In our series, PRES was not fatal and demonstrated excellent clinical reversibility, as indicated by the resolution of the mRS scale. Death and permanent serious neurologic disability have been reported due to progressive cerebral edema and intracerebral hemorrhage. Recurrence is infrequent, occurring in less than 10 percent of the cases [16,17]. The impact of steroid therapy on patient outcomes cannot be addressed in this study and requires robust randomized controlled trials for validation.

The primary strengths of our study include a specific cohort of oncology patients, normotensives, and the validation of MRI findings by both neurological and radiological experts. The limitations of this study encompass the absence of a follow-up MRI, the small sample size of the case series, and the lack of a control group and statistical analysis.

## Conclusions

Posterior reversible encephalopathy syndrome is a neurological emergency that may evade the discerning attention of the attending physician. Owing to the extensive range of clinical features and MRI findings, the list of differential diagnoses is substantial. The reversibility of symptoms, while not always applicable, is predominantly certain, as demonstrated in our case series. We must extend our focus beyond conventional risk variables such as hypertension to consider additional clinical insults. Additional research is necessary about biomarkers for early detection, progress in imaging methodologies, and immunomodulatory treatments, particularly corticosteroids.
